# Analyzing determinants from both compositional and contextual level impeding desired linear growth of children in Indian context

**DOI:** 10.1186/s40795-023-00725-w

**Published:** 2023-06-16

**Authors:** Tamal Basu Roy, Tanu Das, Partha Das, Priya Das

**Affiliations:** Department of Geography, Raiganj University, Uttar Dinajpur, West Bengal Raiganj, India 733134

**Keywords:** Children, Contextual, India, Individual, Multilevel, Stunting

## Abstract

Childhood stunting is recognized as significant public health concern in India. It is a form of malnutrition with impaired linear growth and creates a range of adversaries among children, including under-5 mortality, morbidity, and physical and cognitive growth. The purpose of the present study was to recognize the various leading determinants causing childhood stunting from both individual and contextual level in Indian context. Data were obtained from the India’s Demography and Health Survey (DHS) conducted in 2019–2021. A total of 1, 46,521 children aged 0–59 months were included in this present study. The study applied a multilevel mixed-effect logistic regression model in which individual factors nested within community based contextual-level factors estimating the likelihood of childhood stunting phenomena among Indian children. The variance explained in full model accounted for about 35.8% of the odds of stunting across the communities. The present study elucidates that the recognized factors from individual level characteristics have really increased the odds of childhood stunting: gender of child, multiple births, low birth weight, low BMI among mothers, less educational attainment by mothers, maternal anemic status, breast feeding duration longer than usual, < 4 antenatal care (ANC) visits during gestation period. Similarly, contextual-level factors like rural places of residence, western Indian children, and communities with high poverty rates, lower literacy rates, improper sanitation, and unsafe drinking water were also found to have a significant positive association with childhood stunting. The study finally concludes that cross level interaction between individual and contextual-level factors are identified as significant determinants of linear growth retardation among child in India. In order to reduce this type of malnutrition among the child one should more concentrate on both individual and contextual-level factors as a notable reasons.

## Introduction

Childhood stunting is one of the three anthropometric faces of malnutrition and a serious public health concern. It is an indication of severe, irreversible physical, psychological, and cognitive impairment among children caused by early-life chronic malnutrition. Compared to the developed countries, the increasing trend of childhood stunting is worst in developing countries [1] and India is not an exception. The National Family and Health Survey (2019–21) estimated that approximately 36% of Indian under-five children are suffering from stunting (short height for age). However, the rate of childhood stunting has decreased by 2%, according to NFHS-5 estimates; the phenomenon of childhood stunting is still alarming in India. After the COVID-19 scenario, the wrath of pandemic’s impact on childhood stunting would become terrible and could persist for years, as estimated by UNICEF [2]. According to UNICEF, WHO, and the World Bank Group's joint programme 2021, there are 149.2 million under-five children who are stunted worldwide and constitute 18.95% out of all children. The World Health Organization set a goal in 2012 to reduce the prevalence of stunting by up to 40% by 2025 [3]. In the year 2015, the United Nations Sustainable Development Goals (SDGs) set the goal of “ending all forms of malnutrition globally by 2030” [4]. Despite setting the several worldwide goals for reducing stunting phenomena, India still bears about 30.90% of the total burden of childhood growth retardation as stated by UNICEF [5].

Numerous studies conducted in India at the national and sub-national levels have provided shreds of evidence that a number of factors were associated with childhood stunting. The underlying risk factors which are identified as dominant factors shaping childhood stunting phenomena are the age of the child, male gender [6], higher birth order [7, 8], severe anemia level [9], delayed introduction of breastfeeding [10], small child size at birth [11], unsafe stool disposal practice of child [12], lower maternal age [13], lesser maternal education [14, 15], maternal underweight [13, 16], not attending recommended antenatal care visits [17], maternal short height [18]. Concerning, socio-economic & environmental factors which are comprised with rural setting of residence [19], the Muslim contextual, backward castes [20], households with lower income [21], inadequate or no toilet facilities, unimproved drinking water sources [22] and are highly associated with childhood stunting. An optimum child's growth may be hindered and become irreversibly stunted if they do not receive appropriate nutrition during their first 1000 days of life [23]. Maternal height plays a significant role to avert the stunting phenomena because stunting is a cyclical process, and mothers who themselves were stunted in their childhood are more likely to have stunted children. This leads to a transmission of cycle of poverty from one generation to next generation and diminished human capital which is hard to overcome [24]. Notably, stunting has a strong association with socio-economic & environmental factors of children, as children from low-income families are more likely to be stunted due to lack of improved defecation places, taking unsafe drinking water, as compared to high-income families [22, 25, 26]. Children who have been failure to achieve the ideal height due to linear growth anomaly would ultimately have less working capacity in the near future and lose their capability to work in contrast to other people [15].

There are many earlier researches which have determined the prevalence of childhood stunting and analyzed socio-economic, demographic and cultural factors associated with childhood stunting in India [27–30]. However, most of the existing studies employed a standard binary logistic regression model to identify the individual predictors of childhood stunting. Sometime analysis of individual factors from different levels at one single common level by applying a standard binary logistic regression model leads to biased results (Loss of power or type I error). Furthermore, with the presence of differences in regional settings and other contextual differences in the study of malnutrition becomes complicated. Household and individual level characteristics within same geographic region share common contextual characteristics such as seasonal or climatic variability, types of crops and housing which can have a similar impact on the nutritional status of children within the same cluster. The assumptions of independence among individuals within the similar homogeneous cluster are maintained in hierarchical data structure and sometime equal variance across clusters are violated in the case of cluster arrangement of data or nested data. Hence, the suitable statistical technique for such kind of study must be selected as hierarchical modeling or multilevel analysis. According to Guo and Zhao, multilevel logistic regression analysis has several benefits over traditional logistic regression analysis [30].

Unfortunately, none of the previous studies in the existing literature has highlighted the possible contextual effect of impact of location, homogeneous cluster of socio-economic characteristics and environmental hygiene on childhood stunting in Indian context. In developing countries, the likelihood of the impact of contextual effect on malnutrition is quiet high [31]. From this backdrop, this study aims to identify the significant factors of childhood stunting from both individual and contextual level hierarchical dataset.

### Study design

#### Data

This study utilized data from the 5^th^ round of the National Family Health Survey (NFHS-5), conducted in 2019–2021. This population-based, large-scale, nationally representative sample survey, collected data from all 707 districts in 29 states and 7 Union Territories and gathered information from 636,699 households with a response rate of 98%, while724, 115 women aged 15–49 years with a response rate of 97%, and 101, 839 men aged 15–54 years with a response rate 92%. The information related to child (0–59 months) health indicators were taken from the mothers. The survey was conducted by the International Institute for Population Science (IIPS) under the stewardship of the Ministry of Health and Family Welfare (MoHFW), Government of India. The multi-stage sampling design was adopted to collect information from the respondents. Firstly, the country was divided into rural and urban parts. The rural sample was selected through a two-stage sample design where the villages were treated as the primary sampling units (PSUs) at the first stage (Selected with probability proportional to size), followed by a random selection of 22 households from each PSU at the second stage. In the case of urban areas, there was also a two-stage sampling design with Census Enumeration Blocks (CEB) selected at the first stage and a random selection of 22 households from each CEB at the second stage. In the second stage in both rural and urban areas, households were selected after conducting a complete mapping and household listing operation that was selected at first-stage units.

### Study participants

NFHS-5 collected information for 2, 32,920 children aged 0–59 months. Child-related information was recorded from the response of their mother. Children’s information paired with the mother’s information as individual level factors and location, literacy and poverty rate at a glance and neighbor hood’s hygiene from both PSU and CEB as community level contextual factors was taken in the backdrop of childhood stunting for every child. However, there was missing information for 8,702 children. After removing all missing information and pairing children’s information with mothers' and including contextual information the full information was available for 146,521 children. That’s why the final study participants for the present study were 1, 46,521.

### Outcome variable

Childhood stunting (0–59 months) was considered as the only outcome of interest for the present study. Linear growth retardation of children usually called childhood stunting was measured in height for age during the survey. According to the World Health Organization (WHO) stunting is defined as height for age < -2 standard deviation and severe stunting defined as height for age < -3 standard deviation from the median age (both fall left tail of standard normal distribution) as recommended by WHO in 2006 as child growth standards [32]. In this study children are coded as ‘1’ if they were stunted while not stunted children are coded as’0’. In this study both the stunted and severe stunted children are considered as stunted. Though the indicator was simplistic and the definition reflected on the assessment of the dynamics of growth and could be used consistently over the whole growth trajectory.

### Explanatory variables

Several explanatory variables were included in the present study and considered as potential predictors of childhood stunting. The variables were taken from three different levels i.e. (i) Individual level (compositional variable) (ii) Household level and (iii) Contextual level (regional level). The detailed definition and coding for each explanatory variable have given in Table 1. Here the study used the term contextual level factor to represent the homogeneous clusters within the same geographical living environment and sharing the similar socio-economic conditions. Contextual factors (location, socio-economic condition and environmental hygiene) were chosen based on sharing a common PSU within the DHS data. The clusters were taken as a unit of analysis because of two reasons. First, in the DHS sample, PSUs were used to define the clusters based on homogeneous characteristics. Secondly, it has been shown that most of the DHS is conducted by taking the sample size per PSU that can meet the optimum size with a tolerable precision loss.

**Table 1:** Selection and description of the variables

Variables	Description of the variables
**Individual or compositional -level factors**	
Child's age (Months)	Categorized into (1) 0 – 11; (2) 12 –23; (3) 24 –35; (4) 36– 47; and (5) 48 – 59
Sex of the Child	Categorized into (1) Female and (2) Male
Type of Birth	Categorized into (1) Single and (2) Multiple birth
Birth Weight (grams)	Categorized into (1) Normal ≥ 2500; (2) Low < 2500
**Maternal/Household factors**	
Mother's age during delivery (Years)	Categorized into (1) 15–24; (2) 25 – 34; (3) 35– 49
Mother's Education level	Categorized into (1) No formal education; (2) Primary; (3) Secondary; (4) Higher
Breastfeeding duration (Months)	Categorized into (1) < 6; (2) 6–12; (3) 13–24; (4) > 24
Mother's BMI (Kg/m2)	Categorized into (1) < 18.5; (2) 18.5–24.9; (3) 25.0
Mother's ANC Visits	Categorized into (1) < 4; (2) ≥ 4
Anemia	Categorized into (1) Not anemic; (2) Severe; (3) Moderate; (4) Mild
Ethnicity	Categorized into (1) Others; (2) SC; (3) ST
Sex of the Household Head	Categorized into (1) Male; (2) Female
Wealth Quintile	Categorized into (1) Richest; (2) Poorest; (3) Poorer; (4) Middle; (5) Richer
**Contextual or Regional level factors**	
Place of residence	Categorized into (1) Rural; (2) Urban
Residing region	Categorized into (1) South; (2) North; (3) Central; (4) East; (5) North East; (6) West
Poverty Rate	Proportion of households living below poverty level (wealth index below 20%, poorest quintile).Categorized into (1) Low; (2) High. Median value serves as the reference for the low and high groups
Literacy Rate	Proportion of people in the contextual with no formal education. Categorized into (1) Low; (2) High. Median value serves as the reference for the low and high groups
Proper Sanitation	Categorized into (1) Yes; (2) No
Safe Drinking Water	Categorized into (1) Yes; (2) No

### Statistical analysis

At first univariate analysis was carried out to describe about the participant’s characteristics and after that the bivariate analysis along with Chi-square test statistics was performed to test the association of the sample distribution between the dependent and independent variables. The survey weights were applied throughout the analysis due to the hierarchal nature of the survey and to represent the results in accordance with the country’s population. Finally, a bi-variable and multivariable multilevel logistic regression model was performed to identify the factors that were significantly associated with childhood stunting. For the multilevel analysis, a total of four models were specified in the current study. The first model, commonly known as the empty or null model, was considered without any explanatory variable i.e. simple component or variance analysis. The first model also evaluates the extent of the cluster variation on stunting. The second model namely, the fixed effect model controlled for the individual level variables. The third model also known as the fixed effect model and controlled for the contextual-level variable without considering any individual-level variable and the final model namely, the random slope or random coefficient model controlled for both individual-level variable and contextual-level variables simultaneously. The results of the random slope model were reported as an adjusted odd ratio (aOR) at a 95% confidence interval (95% CI). The intra-class correlation coefficient (ICC) was used to report the variation of stunting at higher contextual level.

### Ethical statement

The ethical approval of the NFHS-5 (2019–2021) was obtained from the ethics review board of the International Institute for Population Science (IIPS), Mumbai. The survey was also reviewed and approved by the ICF and International Review Board (IRB). The study also confirmed that all methods were performed in accordance with the relevant guidelines and regulations. For participation in this survey, informed written and verbal consent was obtained from the participants at the time of the survey. Each participant’s approval was sought, and after that, the interview was conducted. The study involved secondary analysis of publicly available data. Thus independent ethical approval was not needed. However, the corresponding author received permission from www.dhsprogram.com to use the data for analysis.

## Results

### Participant’s characteristics

A total of 146,521 children aged 0–59 months were included in the present study. Of whom, 53.75% were male and 46.25% are females. Among the total, more than 70% of children were single-born babies in a family. Most of the children (84.09%) had a normal birth weight that is more than or equal to 2500 g at birth. More than half of (58.46%) the children’s mother belongs to the age bracket between 25 to 34 years. Nearly 70% of mothers had a formal school education up to the secondary level and above (51.94 + 16.91) secondary level. The breastfeeding duration of the children varied by month, for example, one in every fourth of children had breastfeeding duration of at least 1 year while 23.87% of children had breastfeeding duration of more than two years. More than 60% of mothers had reported their BMI as between 18.5–24.9 kg/m^2^. About 60% of mothers had visited the antenatal clinic at least 4 times or more than that and the remaining were taken antenatal care less than 4 times. Among all mothers, 40% reported that they are not anemic and the rests of the mothers were mild to severely anemic. The majority of the participants (88.25%) were from Scheduled Caste (SC) communities and male-headed families (85.01%). Participants are near about equally distributed in different wealth quintile categories. The majority of the study participants were from rural areas (72.76%). The participant hails from different Indian geographical regions like North (13.77%), South (16.52%), East (26.44%), West (12.83%), and North-East (3.96%) etc. About 23% respondents were from below poverty level and 32.32% mothers were from low literacy rate. About 16.43% respondents use to consume unsafe drinking water. The details of the participant’s characteristics have given in Table 2.

**Table 2:** Socio-demographic and economic characteristics of the respondents

Variables	Frequency	Weighted Percentage
**Individual-level factors**		
**Child's age (Months)**		
0—11	37,386	25.68
12–23	35,594	24.29
24—35	29,402	20.07
36—47	23,666	16.01
48—59	20,473	13.95
**Sex of the Child**		
Male	78,433	53.75
Female	68,088	46.25
**Type of Birth**		
Single Birth	104,550	71.1
Multiple Birth	41,971	28.9
**Birth Weight (grams)**		
≥ 2500	124,590	84.09
< 2500	21,931	15.91
**Maternal/Household factors**		
**Mother's age (Years)**		
15—24	44,733	32.69
25—34	86,460	58.46
35—49	15,328	8.85
**Mother's Education level**		
No formal education	29,322	19.31
Primary	18,034	11.84
Secondary	77,302	51.94
Higher	21,863	16.91
**Breastfeeding duration (Months)**		
< 6	25,489	17.79
06–12	35,770	25.21
13—24	48,793	33.13
> 25	36,469	23.87
**Mother's BMI (Kg/m**^**2**^**)**		
< 18.5	26,813	18.97
18.5 -24.9	92,242	60.91
≥ 25.0	27,466	20.12
**Mother's ANC Visits**		
< 4	60,144	39.99
≥ 4	86,377	60.01
**Anemia**		
Not Anemic	60,560	40.66
Severe	3366	02.15
Moderate	44,379	30.70
Mild	38,216	26.49
**Ethnicity**		
SC	116,452	88.25
ST	22,256	06.35
Others	7813	05.40
**Sex of the Household Head**		
Male	124,512	85.01
Female	22,009	14.99
**Wealth Quintile**		
Richest	21,044	16.75
Poorest	37,189	22.78
Poorer	33,694	21.32
Middle	28,824	19.84
Richer	25,770	19.31
**Contextual level factors**		
**Place of residence**		
Rural	115,687	72.76
Urban	30,834	27.24
**Region**		
South	18,506	16.52
North	27,786	13.77
Central	36,034	26.48
East	28,418	26.44
North East	22,232	03.96
West	13,545	12.83
**Poverty Rate**		
Low	37,189	22.78
High	109,332	77.22
**Literacy Rate**		
Low	47,356	32.32
High	99,165	67.68
**Proper Sanitation**		
Yes	98,614	66.67
No	47,907	33.33
**Safe Water**		
Yes	120,110	83.57
No	26,411	16.43

### Prevalence of childhood stunting

A total of 36% of children aged between 0–59 months were identified stunted from different parts of India. A significantly higher prevalence of childhood stunting was observed among male children (male: 35.50%; female: 32.13%; *P*: < 0.05) in comparison to female children. However, the prevalence of childhood stunting was found equally distributed in different age groups continuing with a certain interval but a significant difference was there. Childhood stunting was slightly higher in the case of multiple births (multiple births: 40.23%; Single birth: 33.88%; *P* < 0.05) and with low birth weight (BW) (BW < 2500:41.65%; BW ≥ 2500: 32.57; *P* < 0.05) babies. The proportion of childhood stunting is significantly highest in children born to mothers with no formal education (42.79%); low Body Mass Index (BMI) (41.52%); surviving with severe anemia (39%); taken less than 4 antenatal care treatment (36.58%) during pregnancy. The prevalence of childhood stunting was significantly highest among children whose parents were from the poorest (42.68%) background with respect to other wealth quintile categories. The proportion of childhood stunting was found higher in rural areas in comparison to urban (rural: 35.32%; urban: 28.47%; *P* < 0.05) counterparts. Prevalence of childhood stunting was also significantly higher in children born to communities with low poverty rate; (42.68%) low literacy rate (41.59%); no proper sanitation (36.56%) and consume unsafe drinking water (34.39%). The detail description about the prevalence of childhood stunting has been given in Table 3.

**Table 3:** Childhood stunting by compositional and contextual level

Variables	Prevalence of Childhood stunting		*P*-Value
	**Stunted (%)** (Weighted 36%)	**Not Stunted (%)** (Weighted 64%)	
**Individual-level factors**			
**Child's age (Months)**			
0—11	24.85	75.15	< 0.001
12–23	39.76	60.24	
24—35	36.74	63.26	
36—47	37.04	62.96	
48—59	32.78	67.22	
**Sex of the Child**			
Male	35.50	64.50	< 0.001
Female	32.13	67.87	
**Type of Birth**			
Single Birth	33.88	66.12	< 0.001
Multiple Birth	40.23	59.77	
**Birth Weight (grams)**			
≥ 2500	32.57	67.43	< 0.001
< 2500	41.65	58.35	
**Maternal/Household factors**			
**Mother's age (Years)**			
15—24	34.41	65.59	< 0.001
25—34	33.53	66.47	
35—49	34.84	65.16	
**Mother's Education level**			
No formal education	42.79	57.21	< 0.001
Primary	39.64	60.36	
Secondary	32.26	67.74	
Higher	23.25	76.75	
**Breastfeeding duration (Months)**			
< 6	26.46	73.54	< 0.001
06–12	29.14	70.86	
13—24	38.41	61.59	
≥ 25	37.87	62.13	
**Mother's BMI (Kg/m2)**			
< 18.5	41.52	58.48	< 0.001
18.5 -24.9	33.91	66.09	
≥ 25.0	26.60	73.40	
**Mother's ANC Visits**			
< 4	36.58	63.42	< 0.001
≥ 4	32.09	67.90	
**Anemia**			
Not Anemic	32.18	67.82	< 0.001
Severe	39.90	60.1	
Moderate	36.24	63.76	
Mild	33.50	66.5	
**Ethnicity**			
SC	33.77	66.23	< 0.001
ST	35.26	64.74	
Others	32.66	67.34	
**Sex of the Household Head**			
Male	33.70	66.3	< 0.001
Female	35.24	64.76	
**Wealth Quintile**			
Richest	22.61	77.39	< 0.001
Poorest	42.68	57.32	
Poorer	37.36	62.64	
Middle	32.64	67.36	
Richer	27.52	72.48	
**Contextual level factors**			
**Place of residence**			
Rural	35.32	64.68	< 0.001
Urban	28.74	71.26	
**Region**			
South	30.90	69.1	< 0.001
North	28.46	71.54	
Central	36.82	63.18	
East	37.34	62.66	
North East	32.90	67.1	
West	36.18	63.82	
**Poverty Rate**			
Low	42.68	57.32	< 0.001
High	30.96	69.04	
**Literacy Rate**			
Low	41.59	58.41	< 0.001
High	30.28	69.72	
**Proper Sanitation**			
Yes	31.20	68.8	< 0.001
No	39.56	60.44	
**Safe Water**			
Yes	31.88	68.12	< 0.001
No	34.39	65.61	

### Compositional and contextual level factors of childhood stunting

Table 4 represents the result of Multi-level model for both individual level and contextual level factors. As mentioned above Model 1 in Table 4 was an empty model while Model 2 & 3 was step wise fixed effect model for individual level and contextual level factors and finally Model 4 represents a complete model adjusted for both individual level and contextual level factors at a time. Children’s age was statically significantly associated with the odds of childhood stunting. Male children were more likely to be stunted compared to the female children (aOR: 1.14; 95% CI: 1.11 – 1.17; *P* < 0.001). Children who were the products of multiple births were 26% more likely to be stunted (aOR: 1.26; 95% CI: 1.14 – 1.41; *P* < 0.001). Children born with low birth weight (< 2500 g) were 44% more likely (aOR: 1.44; 95% CI: 1.39 – 1.54; *P* < 0.001) to be stunted in respect to normal birth weight children. Increases with maternal age and level of education the probability of childhood stunting has decreased gradually. Result revealed that the odds of a child being stunted increases with increasing breastfeeding duration after 6 months. Children of mothers with BMI ≥ 25.0 kg/m^2^were 32% less likely (aOR: 0.68; 95% CI: 0.65 – 0.70; *P* < 0.001) to be stunted compare to the children of mothers with low BMI (< 18.5 kg/m^2^). Children born to mother with more than 4 ANC visits during pregnancy were 10% less likely to be stunted (aOR: 0.90; 95% CI: 0.87 – 0.95; *P* < 0.001). Children born to anemic mothers were more likely to be stunted than the children born to non-anemic mothers. Likewise children born to SC and ST families were more likely to be stunted than the children born to non-SC& ST families. The odds of being stunted decreases with increasing wealth index level. Although these are the factors discussed above were from individual or single level and household context but there has some contextual effects among them because the odd ratios were not identical in fixed effect models and random slope model or adjusted model.

**Table 4:** Model without and with cross level interaction; compositional and contextual level factors with childhood stunting (aged 0–59 Months) in India

Variables	Empty Model		Fixed Effect Model		Fixed Effect Model		Random Slope Model	
	**Model 1**		**Model 2 (Individual)**		**Model 3 (Contextual)**		**Model 4 (Individual &Contextual)**	
	**OR (95% CI)**	***P*—Value**	**aOR (95% CI)**	***P*—Value**	**aOR (95% CI)**	***P*—Value**	**aOR (95% CI)**	***P*—Value**
**Individual-level factors**								
**Child's age (Months)**								
0 – 11			**Ref**				**Ref**	
12–23			1.88 (1.79–1.97)	< 0.001			1.87 (1.79–1.96)	< 0.001
24 – 35			1.67 (1.59–1.75)	< 0.001			1.65 (1.57–1.73)	< 0.001
36 – 47			1.59 (1.51–1.84)	< 0.001			1.53 (1.46–1.82)	< 0.001
48 – 59			1.45 (1.38–1.53)	< 0.001			1.44 (1.37–1.51)	< 0.001
**Sex of the Child**								
Female			**Ref**				**Ref**	
Male			1.18 (1.15–1.21)	< 0.001			1.14 (1.11–1.17)	< 0.001
**Type of Birth**								
Single Birth			**Ref**				**Ref**	
Multiple Birth			1.28 (1.12–1.46)	< 0.001			1.26 (1.14–1.41)	< 0.001
**Birth Weight (grams)**								
≥ 2500			**Ref**				**Ref**	
< 2500			1.50 (1.45–1.55)	< 0.001			1.44 (1.39–1.54)	< 0.001
**Maternal/Household factors**								
**Mother's age (Years)**								
15 – 24			**Ref**				**Ref**	
25 – 34			0.96 (0.93–0.99)	< 0.001			0.95 (0.93–0.99)	< 0.001
35 – 49			0.93 (0.89–0.97)	< 0.001			0.92 (0.89 -0.98)	< 0.001
**Mother's Education level**								
No formal education			**Ref**				**Ref**	
Primary			0.93 (0.89–0.97)	< 0.001			0.94 (0.90–0.98)	< 0.001
Secondary			0.77 (0.74–0.79)	< 0.001			0.77 (0.75–0.80)	< 0.001
Higher			0.73 (0.67–0.81)	< 0.001			0.62 (0.59–0.65)	< 0.001
**Breastfeeding duration (Months)**								
< 6			**Ref**				**Ref**	
06–12			1.02 (0.98–1.06)	0.203			1.02 (0.98–1.05)	0.293
13 – 24			1.21 (1.14–1.25)	< 0.001			1.19 (1.14–1.25)	< 0.001
> 25			1.22 (1.16–1.28)	< 0.001			1.24 (1.18–1.30)	< 0.001
**Mother's BMI (Kg/m2)**								
< 18.5			**Ref**				**Ref**	
18.5 -24.9			0.81 (0.78–0.83)	< 0.001			0.82 (0.79–0.84)	< 0.001
≥ 25.0			0.66 (0.64–0.69)	< 0.001			0.68 (0.65–0.70)	< 0.001
**Mother's ANC Visits**								
< 4			**Ref**				**Ref**	
≥ 4			0.94 (0.92–0.97)	< 0.001			0.90 (0.87–0.95)	< 0.001
**Anaemia**								
Not Anemic			**Ref**				**Ref**	
Severe			1.24 (1.15–1.34)	< 0.001			1.17 (1.15–1.34)	< 0.001
Moderate			1.11 (1.06–1.13)	< 0.001			1.05 (1.02–1.13)	< 0.001
Mild			1.01 (0.98–1.04)	0.456			1.01 (0.97–1.05)	0.458
**Ethnicity**								
Others			**Ref**				**Ref**	
SC			1.11 (1.05–1.18)	< 0.001			1.06 (1.02–1.13)	< 0.001
ST			1.01 (0.94–1.08)	0.692			1.02 (0.96–1.09)	0.421
**Sex of the Household Head**								
Male			**Ref**				**Ref**	
Female			1.04 (1.01–1.07)	0.021			1.11 (1.04–1.18)	< 0.001
**Wealth Quintile**								
Richest			**Ref**				**Ref**	
Poorest			1.95 (1.86–2.06)	< 0.001			1.84 (1.72–1.91)	< 0.001
Poorer			1.68 (1.60–1.76)	< 0.001			1.55 (1.44–1.67)	< 0.001
Middle			1.46 (1.39–1.53)	< 0.001			1.31 (1.11–1.48)	< 0.001
Richer			1.19 (1.14–1.25)	< 0.001			1.18 (1.03–1.36)	< 0.001
**Contextual level factors**								
**Place of residence**								
Urban					**Ref**		**Ref**	
Rural					1.15 (1.11–1.19)	< 0.001	0.94 (0.91–0.98)	< 0.001
**Region**								
South					**Ref**		**Ref**	
North					0.81 (0.77–0.85)	< 0.001	0.82 (0.78–0.86)	< 0.001
Central					1.05 (1.01–1.10)	< 0.001	0.97 (0.92–0.99)	< 0.001
East					1.01 (0.95–1.05)	0.836	0.87 (0.82–0.91)	< 0.001
North East					0.91 (0.86–0.95)	< 0.001	0.81 (0.76–0.86)	< 0.001
West					1.21 (1.13–1.27)	< 0.001	1.11 (1.04–1.17)	< 0.001
**Poverty Rate**								
Low					**Ref**		**Ref**	
High					1.76 (1.73–1.78)	< 0.001	1.68 (1.41–1.79)	< 0.001
**Literacy Rate**								
Low					**Ref**		**Ref**	
High					0.70 (0.68–0.72)	< 0.001	0.53 (0.39–0.61)	< 0.001
**Proper Sanitation**								
Yes					**Ref**		**Ref**	
No					1.17 (1.14–1.20)	< 0.001	1..06 (1.03–1.10)	< 0.001
**Safe Water**								
Yes					**Ref**		**Ref**	
No					1.07 (1.01–1.13)	< 0.001	1.11 (1.02–1.26)	< 0.001

*aOR* Adjusted Odd Ratio, *CI* Confidence Interval, *BMI* Body Mass Index

When all other factors were controlled for in random slope model, contextual-level factors like residential place, geographical location, literacy rate, poverty condition, accompanied with sanitation and drinking water facilities were significantly associated with childhood stunting. Children from rural communities (aOR: 0.90; 95% CI: 0.87 – 0.95; *P* < 0.001) were less likely to be associated with childhood stunting compared to the children from urban communities but the result was different before adjusting with the Individual variables. The odds of being stunted were higher among the children from Western India (aOR: 1.11; 95% CI: 1.04 – 1.17; *P* < 0.001) compared to the children from the South Indian region. Children from communities with high poverty rates were 68% more likely to be associated with childhood stunting. The odds of being stunted were lower (aOR: 0.53; 95% CI: 0.39 – 0.61; *P* < 0.001) among the children who were from the communities with high literacy rates compared to the children belonging to the communities with low literacy rates. The existence of improper sanitation and unsafe drinking water facilities within the community were also significantly and positively associated with childhood stunting in India.

### Proportion of total variance of stunting phenomena experienced on contextual level

As shown in Table 5, with respect to the empty model there was a significant variation in the odds of childhood stunting (variance: 0.416; *P*: < 0.001). The ICC pointed out that the estimated intercept component variance showed that an 11.5% variance in the odds of childhood stunting could be attributed to contextual-level determinants. However, after inclusion of contextual level determinants (11.5–5.2) 6.3% variation still exists due to any other contextual level determinants. The variation of childhood stunting across clusters remained statistically significant, even after controlling for individual-level and contextual-level factors (random slope model of the final model). As determined by the percentage change in variance of childhood stunting, the full model or random slope model accounts for about 35.8% of the odds of childhood stunting across the communities. The stepwise decreasing nature of DIC (-2 Loglikelihood) represents that the entire model has improved subsequently and represents a statistically significant model (Model 1 DIC: 92,826.87; Model 4 DIC: 86,153.18).

**Table 5:** Measures of variation and model fitting statistics

Measures of variation	Model 1	*P*-Value	Model 2	*P*-Value	Model 3	*P*-Value	Model 4	*P*-Value
**Contextual level**								
Variance (SE)	0.416 (0.013)	< 0.001	0.226 (0.010)	< 0.001	0.196 (0.019)	< 0.001	0.14 (0.016)	< 0.001
Explained Variation (%)	Reference		35.7		33.4		35.8	
ICC (%)	11.5		8.8		7.3		5.2	
**Model fitting statistics**								
DIC (-2 Log likelihood)	92,826.87		89,278.84		88,142.42		86,153.18	

*SE* Standard Error, *ICC* Intra cluster correlation, *DIC* Deviation information Criteriation; Model 1 is the empty model, a baseline model without any determinant variable; Model 2 is adjusted for individual-level factors; Model 3 is adjusted for contextual-level factors; Model 4 is final model adjusted for both individual- and contextual-level factors

## Discussion

Using data from nationally representative surveys, the study analyzed individual or single level factor from children, mothers and household level and contextual-level factors from the homogeneous areal PSU unit considering location, literacy and poverty as well as neighborhood’s hygiene as significant predictors estimating childhood stunting. It elaborates the significance of contextual-level variability in childhood stunting with respect to individual-level variability. About 36% of the variations (35.8%) in stunting were explained by individual and contextual-level characteristics in the final model. Children between the ages of 12 months and 23 months bear the highest risk of childhood stunting, which usually decline after 23 months. This finding has been validated by previous findings from other developing countries [32–34] including India. It indicates that children's height rapidly decreased from the time of birth to 23 months; however, stunting processes continued after 23 months, but at a considerably slower rate. This might be due to weaning and decreased breast milk intake and not enough supplementary dietary intakes in terms of frequency and variety which predisposes them to childhood stunting [15]. According to the study's findings, stunting in children under the age of five is strongly influenced according to child's sex. Although some of the earlier research demonstrated that, there is no valid link between a child's sex and childhood stunting. While some research revealed that stunting was more prevalent in male children; on the other hand few research also discovered that stunting was more common in female children. However, a recent systematic review and meta-analysis from South-East Asian countries revealed that in the majority of South-East Asian countries, male children were more likely to be stunted compared to female children [6]. Results from this recent study are consistent with those from earlier research [8, 25], which found that compared to single births, children belonging to the multiple birth cohort group are more likely to be stunted. Such kind of linear growth retardation in accordance with age are usually linked to poor nursing, low birth weight, and due to lack of sufficient nutritional intake, all of which are usually happened to multiple-birth children than in single-birth children. The results of this study corroborate those of related studies that found that mother’s educational attainment plays a crucial role as protective factor against childhood stunting [14, 15]. Mothers with formal education may be aware of what to door to avoid childhood stunting or to minimize its severity if it already exists. Desired antenatal care visits during the gestation period are really essential for both women and child and it was really consistently associated with childhood stunting. The risk of stunting was higher in children whose mothers had taken fewer than at least four professional prenatal care visits during pregnancy. This result of our study is consistent with previous finding [17, 35]. Children from the women were less likely to be malnourished if they had taken at least four ANC visits, and was able to discuss pregnancy and childbirth-related matters as well as optimum growth of foetus with professionals and their husband [35]. In contrary, those women who hardly followed any antenatal care visit because of lesser educational attainment and low household income, the foetus is more likely to be malnourished and the newborn offspring is more likely to be stunted as they are deprived of all the prenatal maternal precaution. This study found a substantial correlation between childhood stunting and mother's BMI, indicating that children under five whose mothers had low BMI are more likely to be prone to childhood stunting. A similar finding has been documented in some studies [14, 15]. To the best of our knowledge, we were unable to discover any literature to support the claim that the mother's height may selected as crucial measurement for evaluating this association. In this study, prolonged breastfeeding, especially after infancy (> 12 months), was found as pertinent one of childhood stunting. This conclusion may be explained by the fact that most households in developing nations are underprivileged and unable to provide their children with a sufficient diet of complementary foods of high quality. As a result, they continue to breastfeed exclusively for longer than 12 months without adding any additional foods [36–38]. Another theory is that some infants who are breastfed for longer than usual periods of time reject diets other than breast milk, as demonstrated by a Ghanaian study [39]. Additionally, this study's findings also revealed that under-five children from less affluent households have a higher likelihood of being stunted than under-five children belonging from wealthy households. Similar findings from earlier studies conducted in other developing nations provide more evidence that poverty is a strong predictor of childhood stunting [15, 18, 31, 40]. The socioeconomic condition of a household is important since it frequently affects the availability of healthy foods for children's growth and development. Additionally, under-fives from lower-income homes in the majority of developing nations, like India, where a publically funded health care system is not practiced, would not have access to quality and essential health care when they became unwell. The study revealed that maternal anemia significantly associated with childhood stunting. This could be due to children of anemic mothers experience slower growth because of their moms' severe reduction in working ability, which makes it difficult for them to care for their children and do household duties [22]. Therefore, when anemic moms are unable to provide adequate attention and care owing to own poor health, the harmful effects of maternal anemia that also impact on the infant's linear growth retardation expand and intensify.

The multilevel technique used in this study creates an opportunity to identify also the contextual level effects (area or neighborhood factors) rather than only compositional level effects (individual characteristics effects) entirely to account for variations in childhood stunting among different communities or clusters. Despite this, it has been discovered that differences in individual or single level characteristics account for the majority of the differences in childhood stunting rather than difference between various contextual characteristics. Moreover, the study also declared that contextual or regional characteristics also play a pivotal role in growing childhood stunting phenomena. These results are in line with the majority of research efforts to separate compositional effects from contextual effects [40–42]. The approach of multilevel analysis can help to avoid the traditional problem in single level analysis of likelihood of childhood stunting because it has aggregated individual data to the group level to conduct a group level analysis or disaggregated the group level data to conduct the individual analysis. Therefore, the analysis of likelihood of outcome of childhood stunting might be rational through compromise of both single or individual level and contextual level factors. Hence, the changing odds in the final model also signify the contextual-level factors, on their own, exert a significant influence on individual-level factors.

## Conclusion

The study's conclusions have some important policy ramifications. Intervention is urgently required in lessening economic disparities, which will ultimately lead to poverty among the population. In this regard both short and long term approaches are required. Adult literacy programmes should be expanded across the communities, prioritizing the objectives to enhance the awareness regarding adequate nutritional requirement for both maternal and children’s health. As a long-term strategy, governments of developing nations should also make sure that women from illiterate communities receive a suitable formal education. All these footsteps may consider as an effective effort to enhance the awareness of women from different groups that they can better nourish their children during childhood period. Places, marked with a high rate of childhood stunting should get the priority to initiate any governmental and non-governmental organization’s project that aims to improve the nutritional status of children under-five years ultimately would brought the entire regional level within its ambit. The establishment of nutritional intervention programmes, such as the therapeutic feeding and nursery school feeding programmes, should be prioritized with a focus on higher risk groups, such as male children under the age of five, children with uneducated or less educated mothers, children of multiple births, and children who live in areas with a high illiteracy rate. Those children who are currently experiencing childhood stunting, the necessary interventions are required to minimize the severity of linear growth retardation. Considering and minimization of public service fees, particularly for health care, at the behest of less affluent members of society who are unable to pay for essential health care services will facilitate in reducing the severity of childhood stunting in the patients. Moreover, provision of free health care for pregnant mothers, new mothers and young children need to be carried out uninterruptedly. Finally, it is important to perform future studies that will take into account morbidity variables as well as causal linkages.

## Data Availability

Data have been collected from the portal of Demography and Health Surveys (DHS) that provides data in the areas of population, health and nutrition which is publicly available and anyone can access with a prior request and subject to non-profit and academic interest only. The following data link has been used to extract the data—https://dhsprogram.com/data/dataset_admin/login_main.cfm. The data may be made available upon reasonable request from the corresponding author.

## Abbreviations

ANC: Antenatal Care
BMI: Body Mass Index
BW: Birth Weight
CEB: Census Enumeration Block
DHS: Demographic and Health Survey
ICC: Inter Class Correlation
IIPS: International Institute for Population Science
MoHFW: Ministry of Health and Family Welfare
PSU: Primary Sampling Unit
